# Supplementary material for: A Systematic Review of Individualized Heart Surgery with a Personalized Prosthesis
Source: J Pers Med. 2023 Oct 11;13(10):1483. doi: 10.3390/jpm13101483 (PMC10608049; doi:10.3390/jpm13101483)
Supplement: Supplementary file 1 [file jpm-13-01483-s001.zip › jpm-2587674-supplementary.pdf]

Supplementary Materials

Table S1. Risk of Bias analysis by using GRADEpro.

Study/Year	Risk of Bias	Incon-sistency	Imprecision	Indirectness	Publication Bias	Certainty of the evidence (GRADE)
Amerini et al, 2014 [30]	Not serious	Not serious	Not serious	Not serious	Undetected	⊕⊕◯◯ LOW
Collis et al, 2018 [31]	Not serious	Not serious	Not serious	Not serious	Undetected	⊕⊕◯◯ LOW
Ovcharenko et al, 2016 [32]	Not serious	Not serious	Not serious	Not serious	Undetected	⊕⊕◯◯ LOW
Pasta et al, 2020 [33]	Not serious	Not serious	Not serious	Not serious	Undetected	⊕⊕◯◯ LOW
Rim et al, 2015 [34]	Not serious	Not serious	Not serious	Not serious	Undetected	⊕⊕◯◯ LOW
Robinson et al, 2018 [354]	Not serious	Not serious	Not serious	Not serious	Undetected	⊕⊕◯◯ LOW
Yuan et al, 2017 [36]	Not serious	Not serious	Not serious	Not serious	Undetected	⊕⊕◯◯ LOW

NB: ⊕⊕◯◯ LOW, indicates having the least bias, and results are considered valid.
